# Medical Students’ Perceptions of Point-of-Care Ultrasound Education: A Qualitative Interview Study

**DOI:** 10.7759/cureus.108659

**Published:** 2026-05-11

**Authors:** Grace Nguyen, Parker Nguyen, Shawn Sethi, McKenzie Hollon

**Affiliations:** 1 Anesthesiology, Emory University, Atlanta, USA; 2 Physical Medicine and Rehabilitation, NewYork-Presbyterian Hospital, Columbia and Cornell Department of Physical Medicine and Rehabilitation, New York, USA; 3 Emergency Medicine, Duke University, Durham, USA

**Keywords:** curriculum development, medical student education, point-of-care ultrasound (pocus), qualitative research, undergraduate medical education

## Abstract

Background

Point-of-care ultrasound (POCUS) training is a critical component of undergraduate medical education (UME) due to its application across specialties. However, the best strategy for its integration into UME remains uncertain. Despite growing expert consensus on POCUS competency milestones, the perspectives of learners have received limited attention. This qualitative study explores medical students’ experiences, confidence, and perceived training gaps regarding POCUS training to generate insights to complement expert frameworks and inform curriculum development.

Methods

We conducted interviews with 13 final-year medical students at a single U.S. MD school. Interviews were analyzed using inductive thematic analysis to identify themes related to their POCUS education experiences, instructional preferences, and perceived training gaps. Reporting followed the Consolidated Criteria for Reporting Qualitative Research (COREQ) guidelines.

Results

Three key themes emerged: (1) Students universally valued POCUS training and recognized its importance for residency preparation, but felt inadequately trained. (2) Inconsistent and informal exposure to POCUS limited skill development and confidence, with training opportunities varying by rotation and individual attending interest. (3) Students preferred a structured hybrid teaching model. They expressed a desire to prioritize practical skills like probe selection, machine operation, and focused exams.

Conclusions

Students reported variable POCUS exposure and limited confidence despite recognizing its clinical value. These findings suggest that structured, high-yield POCUS education centered on foundational skills may improve learner engagement and confidence. These findings identify areas where learner experience may differ from existing competency frameworks and may help inform the implementation of introductory UME POCUS curricula.

## Introduction

The integration of point-of-care ultrasound (POCUS) across a growing number of medical specialties has rapidly expanded over the last decade. The traditional role of ultrasound within radiology, obstetrics, and cardiology has expanded to include applications in other specialties, most notably emergency medicine, intensive care, surgery, and anesthesiology [1]. This rapid growth can be attributed to the tool’s usefulness in clinical screening, diagnosis, and management, combined with its easily accessible, portable, and cost-effective nature [2]. Some of its diverse uses include assessment of cardiac structure and function, Focused Assessment with Sonography for Trauma (FAST), and procedural guidance to assist in nerve blocks and vascular access.

Medical schools have begun to implement POCUS into undergraduate medical education (UME) curricula, yet the structure and level of training vary greatly across institutions, leaving significant disparities in proficiency among new residents [3,4]. There is currently no standardized consensus on the POCUS milestones students should achieve before graduation. Efforts to standardize have largely drawn on expert consensus. Dinh et al. established 90 core clinical ultrasound milestones through a modified Delphi survey of ultrasound education directors [5]. Hoppmann et al. published international consensus recommendations for UME POCUS curricula based on input from educators across multiple specialties [6]. These frameworks guide curriculum developers but reflect the priorities of experienced clinician-educators rather than the experiences and perceived needs of the learners themselves.

This gap is important for practical reasons. Curriculum implementation research demonstrates that learner engagement and perceived relevance are important determinants of educational effectiveness [7]. A POCUS curriculum built entirely around expert-defined milestones, without consideration of how students experience their training, could risk poor engagement and inadequate skill acquisition regardless of content quality.

To date, the medical student perspective on POCUS education has been explored primarily through broad attitudinal surveys. Chilstrom et al. surveyed 94 final-year medical students and found near-universal agreement that more ultrasound should be incorporated into the curriculum [8]. While these findings confirm general enthusiasm, they do not provide the nuance needed to inform specific decisions about curriculum content, instructional methods, or timing.

Therefore, we conducted qualitative semi-structured interviews to explore medical students' views on the current state of their UME POCUS training. We sought to understand students’ attitudes toward POCUS training, their confidence in performing POCUS exams, the instructional methods they found most and least effective, and the specific skills they considered highest priority for their transition to residency. Our aim was not to propose a definitive curriculum but to generate learner-derived insights that can complement existing frameworks and inform the design of UME POCUS programs. This study is exploratory and intended to generate hypotheses for future curricular research, not to propose a definitive POCUS curriculum.

## Materials and methods

We conducted a qualitative semi-structured interview study and report our findings according to the Consolidated Criteria for Reporting Qualitative Research (COREQ) checklist (Appendix 1) [9]. A constructivist approach guided the study design, seeking to understand participants’ experiences with POCUS education from their own perspective. This human subject research protocol was submitted to and reviewed by the Emory IRB and approved for exemption (Study #00007936).

Recruitment

Participants were medical students enrolled at Emory University School of Medicine, Atlanta, Georgia. All participants were final-year students in the Doctor of Medicine (MD) program. Each participant had completed the same undergraduate medical education curriculum up until the point of the interview. The participants were sampled from a pool of 140 students. All students were recruited via email. Before the interview, participants were informed that the study aimed to explore medical students' opinions and attitudes regarding their POCUS training. A reminder email was sent to all students who did not respond two weeks after the initial contact. The 13 respondents (n = 13) provided verbal and written consent to participate in the study.

We employed a convenience sampling strategy. The response rate was 9.3% (13/140). We acknowledge that respondents may represent students with strong opinions about POCUS education, which could introduce self-selection bias. This is addressed further in the Limitations section.

Interview format

Interviews (n = 13) were conducted by GN, a female medical student at Emory University School of Medicine. GN had no prior formal training in qualitative research methods but did receive mentorship from one of the senior authors (MH) on interview technique and qualitative study design before data collection. As a peer of the participants and a final-year medical student with experience navigating the same POCUS curriculum, GN brought familiarity with the training environment. While this may have encouraged more candid responses, it also introduced the potential for shared assumptions to go unexamined. To mitigate this, GN followed an interview guide and avoided leading questions. All interviews were conducted over the telephone. No one other than the interviewer and participant was present during interviews. No repeat interviews were conducted. Interview length ranged from 10 to 30 minutes. The interviewer asked each participant a standard set of questions informed by the literature on the topics of demographics, level of exposure, general attitudes, instruction method and timing, and content (Appendix 2). Interviewees were encouraged to reflect on their responses and to offer their own opinions freely.

Data collection and analysis

All interview audio was recorded using the Voice Memo (Apple Inc., Cupertino, USA) application. The audio was then transcribed verbatim using the Descript platform (Descript, Inc., San Francisco, USA). All audio files, transcripts, and data were stored on a secure OneDrive (Microsoft Corporation, Redmond, USA) with access limited to the study team. Field notes were not systematically collected during or after interviews. An inductive thematic analysis was performed on the data according to protocols described by Vears et al. [10]. The analysis was performed by GN and PN, student members of the team. Coding was performed manually using Microsoft Excel (Microsoft Corporation, Redmond, USA; no dedicated qualitative analysis software was used. The transcripts were first read comprehensively before beginning the process of selecting and labeling portions of the text. All potential identifying information was removed. In the first round of coding, sections of the text were identified and labeled based on their “big picture meaning,” and broad categories of content relevant to the research question were identified. During the second round of coding, the text within the broad sections was further coded into subcategories. In the third round of coding, the subcategories created in the previous round were combined or separated to further refine the text into fine-grained subcategories. The result of these three rounds of coding was a refined coding schema. The categories were then analyzed to provide an interpretation that accurately encompassed the interview transcripts. Analysis was performed separately by each researcher. The two researchers’ codes and subcategories were cross-referenced to find consensus. Discrepancies were discussed and resolved through iterative dialogue until agreement was reached. Formal intercoder agreement statistics were not calculated; consensus-based reconciliation was used, given the inductive and interpretive nature of the analysis. A senior member of the research team (MH) reviewed the final coding schema for coherence and completeness.

Although formal data saturation was not assessed using a predetermined threshold, the research team observed that no substantively new themes emerged in the final interviews. This suggests that adequate thematic coverage was achieved for this study, though we do acknowledge that a larger sample size may have yielded additional perspectives. Transcripts and findings were not returned to participants for member checking, which may limit the confirmability of our findings.

## Results

Participant demographics

Thirteen final-year medical students participated in semi-structured interviews. Participant demographics, including sex, age range, intended specialty, and prior POCUS exposure, are summarized in Table 1. Three key themes emerged from the analysis, each with associated subthemes. Quotations presented below were drawn from across the full sample to ensure a broad representation of participant perspectives. Table 2 provides a summary of the three themes, their associated subthemes, and representative quotations.

**Table 1:** Participant Demographics Demographic and educational characteristics of the 13 final-year medical students who participated in semi-structured interviews. Percentages may not sum to 100 due to rounding. POCUS: point-of-care ultrasound.

Characteristic	n (%)
Total participants	13 (100)
Sex	
Female	8 (61.5)
Male	5 (38.5)
Age, years	
Range	25–36
Year of medical school	
Final-year (M4)	13 (100)
Intended specialty	
Internal Medicine	6 (46.2)
Anesthesiology	3 (23.1)
Orthopedic Surgery	2 (15.4)
Pediatrics	1 (7.7)
General Surgery	1 (7.7)
Prior ultrasound experience before medical school	
None	13 (100)
Preclinical POCUS exposure	
No exposure	9 (69.2)
One-time hour-long workshop	4 (30.8)

**Table 2:** Summary of Themes, Subthemes, and Representative Quotations Quotations were drawn from across the full sample of 13 participants to ensure broad representation. POCUS: point-of-care ultrasound.

Theme	Subtheme	Representative Quotation
Theme 1: Universal Recognition of POCUS Value Despite Perceived Inadequacy of Training	Value of POCUS for residency preparation	“Getting exposure before residency is very important. It’s such a big thing across so many specialties, so it’d be nice to at least be familiar with the skill.”
	Low confidence in independent POCUS performance	“I don’t feel like I would be very confident doing an ultrasound on my own the first time.”
Theme 2: Inconsistent and Informal Exposure Limited Skill Development	Preclinical and clinical exposure	“My exposure was very sporadic and really didn’t build into anything until the consistency started with more structured fourth-year rotations.”
	Reliance on informal bedside teaching	“It was really just teaching at the bedside. In theory, it’s like a see one, do one, but most of the time I wouldn’t really get hands-on practice because of time or inconvenience to the patient.”
Theme 3: Preferences for Structured, Hybrid POCUS Education	Preferred teaching methods	“I think what’s missing is we’ve never all had a formal lesson on the basics. It would have been nice to have been shown how to use the machine, what general structures like air versus fluid look like, and how to orient yourself when looking at the screen.”
	Preferred timing of instruction	“The only way that I’d actually get anything from ultrasound sessions would be to have spaced repetition of the skill. Right now, the exposure I’ve gotten is so inconsistent that every time ultrasound is taught again, I feel like I’m starting from scratch.”
	Priority content areas	“Just the basics like what the buttons on the ultrasound machine do, the difference between the probes, and identifying basic structures like the liver and lungs.”
	Perceived barriers to curriculum integration	“The biggest problem is probably limited access to machines and time. It’s not realistic to get sufficient practice as a student because the machines are usually being used for immediate patient care, not teaching.”

Theme one: universal recognition of POCUS value despite perceived inadequacy of training

All students acknowledged the importance of POCUS during medical school and the value that it brings for preparing them for residency. Despite this universal recognition, students consistently reported feeling inadequately prepared to use POCUS independently upon entering residency.

“Getting exposure before residency is very important. It’s such a big thing across so many specialties, so it’d be nice to at least be familiar with the skill.”

Students expressed a range of comfort levels in their ability to interpret POCUS images and perform POCUS exams. There was an inconsistency in the type of POCUS exams students felt most comfortable performing. Most students felt they lacked the necessary experience to perform POCUS exams independently, often describing their confidence as minimal.

“I don’t feel like I would be very confident doing an ultrasound on my own the first time.”

“I feel pretty comfortable with identifying heart failure signs like volume status, but with other things like the FAST exam, I know almost zero.”

Several students acknowledged that confidence and skill in POCUS come with practice, recognizing that their current abilities may develop over time.

“I find it difficult to obtain the images myself, but I’m hoping that just comes with practice.” 

Theme two: inconsistent and informal exposure limited skill development

Preclinical and Clinical Exposure

As shown in Table 1, no participants had exposure or training in POCUS before medical school. During their preclinical curriculum, most students had no exposure to POCUS, while a minority participated in a one-time hour-long workshop. Training during clinical rotations relied heavily on a resident's or attending’s interest.

“My exposure was very sporadic and really didn’t build into anything until the consistency started with more structured fourth-year rotations.”

“I was lucky that I got some attendings that taught ultrasound more than normal in the third year, but I know that’s not the case for most students.”

Reliance on Informal Bedside Teaching

Most of the current POCUS teaching that occurs during clinical rotations is informal teaching at the bedside rather than structured formal learning.

“It was really just teaching at the bedside. In theory, it’s like a see one, do one, but most of the time I wouldn’t really get hands-on practice because of time or inconvenience to the patient.”

“It’s always been relatively informal for me. Maybe with residents and attendings performing ultrasounds on patients and just pointing things out.”

Theme three: preferences for structured, hybrid POCUS education

Preferred Teaching Methods

An overwhelming majority of students expressed a preference for a hybrid of didactic lecture and hands-on practice to learn POCUS. Students valued having foundational knowledge before hands-on sessions and emphasized the importance of low-pressure, non-clinical practice environments.

“I think what’s missing is we’ve never all had a formal lesson on the basics. It would have been nice to have been shown how to use the machine, what general structures like air versus fluid look like, how to orient yourself when looking at the screen, etc. I was sort of just thrown into the clinical environment and seeing ultrasounds performed without any framework of the basics.”

“I’d like it if we had more independent hands-on time to troubleshoot by ourselves in a low-pressure environment. Maybe practicing with classmates in Objective Structured Clinical Examination (OSCE) suites like we did when we learned the physical exam.”

Preferred Timing of Instruction

There were mixed opinions on the best time to introduce POCUS teaching into the curriculum. The majority of students believed POCUS theory should be taught in preclinicals with consistent hands-on training starting during clinical years. A unifying theme was the desire for consistency and repetition.

“The only way that I’d actually get anything from ultrasound sessions would be to have spaced repetition of the skill. Right now, the exposure I’ve gotten is so inconsistent that every time ultrasound is taught again, I feel like I’m starting from scratch. I’d like to have hands-on workshops during specific modules in preclinicals, like cardiology, and then have lessons consistently during M3 clerkships.”

Not all students agreed. Some students believed that POCUS teaching should be introduced strictly in clinical years of medical school.

“I think ultrasound should be strictly taught during the fourth year because students are already learning so much during the first three years of medical school. I think it makes most sense to teach it in a fourth-year elective to help with the transition to residency.”

Priority Content Areas

Students believed the important POCUS skills to learn before graduating medical school included how to use the machine, when to use different probes, how to orient oneself when obtaining images, how to find major organs, and being able to identify common critical findings. They expressed that these skills are manageable for a medical student at their level, but are also clinically important.

“Just the basics like what the buttons on the ultrasound machine do, the difference between the probes, and identifying basic structures like the liver and lungs.”

“Definitely the FAST exam.”

Perceived Barriers to Curriculum Integration

Students identified practical barriers to integrating POCUS education into the existing curriculum, including limited access to machines, time constraints, and questions about relevance across specialties.

“The biggest problem is probably limited access to machines and time. It would be really helpful if students had the opportunity to practice more often, but there are a limited number of machines in the hospital. It’s not realistic to get sufficient practice as a student because the machines are usually being used for immediate patient care, not teaching.”

“Even though I’m really interested in ultrasound, I don’t know how necessary it is for someone who’s going into a specialty that doesn’t really use it, like psychiatry or ophthalmology. It might be hard to get some students to care about ultrasound.”

## Discussion

The results of this study highlight several important themes regarding medical students’ attitudes, confidence, preferences, and perceived barriers regarding learning POCUS. Taken together, these findings offer learner-derived insights that can complement the expert-driven frameworks currently guiding UME POCUS curriculum development.

Value recognition and the confidence gap

Our participants universally recognized POCUS as valuable for residency preparation, consistent with the growing expectation that graduating medical students possess basic POCUS competency [11]. Yet this recognition was accompanied by low confidence in independent POCUS performance. Students described their abilities as “minimal” and expressed discomfort with exams they had received little formal training in, particularly the FAST exam. Confidence was closely tied to hands-on experience, consistent with the broader literature demonstrating that formal POCUS workshops and structured training improve student confidence and skill acquisition [1,12,13]. The inconsistency in which exams students felt comfortable with suggests that confidence tracks specific exposure rather than general aptitude, reinforcing the need for a curriculum that ensures uniform coverage of core applications.

The problem of inconsistent, informal exposure

Perhaps the most striking finding was the degree to which POCUS exposure depended on chance: which rotation a student was assigned to, which attending happened to be on service, and whether that attending had a personal interest in teaching POCUS. Students who had encountered even brief structured training expressed a greater sense of preparedness, underscoring that the issue is not one of student motivation but of curricular design.

The reliance on informal bedside teaching is a particular concern. While bedside instruction offers the advantage of clinical context, prior research suggests it is only effective when students actively participate - something that is difficult to ensure in busy clinical environments [14]. Our participants described a pattern of passive observation rather than hands-on engagement, reinforcing the argument for protected, structured POCUS practice time outside of clinical workflows.

Instructional preferences: the case for a hybrid model

Students overwhelmingly preferred a hybrid model combining didactic instruction with supervised hands-on practice in low-pressure, non-clinical settings. These preferences are consistent with the characteristics of effective POCUS curricula described by Martin et al., who found that programs featuring structured small-group learning, dedicated didactics, and mandatory hands-on practice yielded the greatest improvements in student skills, confidence, and understanding [15]. The convergence between learner preferences and evidence-based best practices strengthens the case for the hybrid approach, though it is worth noting that student preferences do not always align with evidence-based teaching methods. The value of these findings lies not in treating student preferences as prescriptive but in understanding them as one data point alongside empirical evidence and expert judgment.

Timing of POCUS instruction

Timing was the area of greatest disagreement among participants. Despite this disagreement, a unifying theme emerged: students wanted consistency and repetition regardless of when training began. Multiple participants described a pattern of sporadic exposure followed by long gaps, leaving them feeling like they were “starting from scratch” each time. Martin et al. demonstrated that preclinical and clinical training phases offer distinct benefits, and standalone workshops boost confidence, though longitudinal curricula tend to yield better retention [15,16]. Time constraints remain a significant barrier, and the optimal timing of POCUS education likely varies by institutional context [17,18].

Learner-perceived foundational priorities in UME POCUS

A notable finding was the gap between what students viewed as achievable during medical school and the milestones established by expert consensus. Our participants prioritized foundational skills: machine operation, probe selection, image orientation, identification of major organs, and recognition of critical findings on a FAST exam. By contrast, expert frameworks such as the 90 milestones proposed by Dinh et al. set considerably more ambitious targets [5].

This disconnect raises two questions: are the expert-defined milestones realistic, given the current state of POCUS education at most institutions, and could their ambitious scope inadvertently discourage institutions from implementing any structured curriculum at all?

We do not suggest that student preferences should override expert judgment. However, our findings generate a testable hypothesis: that a focused UME POCUS curriculum centered on machine operation, probe selection, FAST, and basic cardiac views may represent a feasible and effective starting point for institutions building or expanding their POCUS curricula. Future intervention studies comparing focused versus comprehensive curricula on residency performance outcomes would help resolve this question.

Practical implications for curriculum developers

While this exploratory study cannot prescribe a specific curriculum, several practical observations emerge. Students consistently identified the lack of foundational instruction as the most significant gap, suggesting that even a single structured session before clinical rotations could make subsequent clinical exposure more productive. The dependence on individual attending interest argues for embedding structured POCUS sessions within clerkship curricula rather than relying on opportunistic bedside teaching. Students valued low-pressure practice environments such as simulation labs and OSCE suites, particularly early in training. Finally, the concept of spaced repetition emerged organically from interviews, suggesting that distributing POCUS concepts across the curriculum may improve retention more than concentrated but infrequent training blocks. These observations are hypothesis-generating rather than prescriptive. Their implementation would need evaluation through future curricular intervention studies.

The role of learner perspectives in curriculum design

A potential criticism is that student preferences are an unreliable guide to curriculum design. We acknowledge this limitation. However, learner perspectives serve a different function than expert recommendations: where expert frameworks define what should be taught, learner perspectives reveal where training breaks down in practice and where engagement is highest. This approach has precedent in medical imaging education. Harthoorn et al. used qualitative interviews with students and residents to explore perspectives on radiology education [19]. Our study extends this line of inquiry by systematically interviewing a larger cohort and analyzing their responses through a structured thematic framework.

Future directions

Several directions for future research emerge from these findings. Multi-institutional studies would help determine whether the themes identified here are generalizable. Studies incorporating residency program directors’ perspectives on expected POCUS competencies would provide an important complementary viewpoint. Intervention studies comparing focused versus comprehensive curricula on measurable residency performance outcomes would help resolve the content prioritization question. Finally, longitudinal studies tracking whether UME POCUS training translates into improved clinical performance during residency remain an important gap.

Limitations

This study has several limitations. The sample size of 13 students from a single institution limits generalizability. The response rate of 9.3% introduces self-selection bias, as respondents may hold stronger views about POCUS education than non-respondents. The study was conducted at a single U.S. MD school with a specific curricular structure; students at institutions with more established POCUS programs may have different experiences. Nearly half of the participants intended to pursue Internal Medicine, a specialty where POCUS is increasingly perceived as clinically relevant, which may have biased findings toward greater enthusiasm for ultrasound education than would be observed in a more specialty-diverse sample. The interviewer (GN) was a peer of the participants, which may have introduced shared assumptions, though steps were taken to mitigate this through a standardized interview guide. Member checking was not performed, which may limit the confirmability of our interpretations. Finally, as an exploratory qualitative study, these findings are hypothesis-generating rather than definitive; they identify themes and patterns that warrant further investigation through larger, multi-institutional studies with measurable outcomes.

## Conclusions

Medical students in this study universally valued POCUS education but felt inadequately prepared by their current training, which was characterized by inconsistent, informal, and attending-dependent exposure. They preferred a hybrid instructional model combining didactic foundations with hands-on practice in low-pressure settings, and they emphasized foundational competencies such as machine operation, probe selection, and focused applications as realistic pre-graduation targets. These learner perspectives do not replace expert judgment but offer a complementary lens for curriculum design - one that highlights where training breaks down in practice and where student engagement is likely to be highest. As institutions continue to develop and refine UME POCUS programs, incorporating the experiences of learners alongside expert recommendations may help produce curricula that are both educationally sound and responsive to how training is experienced in practice.

## Appendices

Appendix 1 

**Table 3:** Consolidated Criteria for Reporting Qualitative Research (COREQ): 32-Item Checklist POCUS: point-of-care ultrasound.

#	Topic	Guide Question / Description	Response (page # or description)
Domain 1: Research Team and Reflexivity			
Personal Characteristics			
1	Interviewer	Which author(s) conducted the interviews?	GN (Methods: Interview Format)
2	Credentials	What were the researcher’s credentials?	Medical student, MD candidate
3	Occupation	What was their occupation at the time?	Final-year medical student (Methods: Interview Format)
4	Gender	Was the researcher male or female?	Female (Methods: Interview Format)
5	Experience and training	What experience or training did the researcher have?	No formal qualitative training (Methods: Interview Format)
Relationship with Participants			
6	Relationship established	Was a relationship established before the study?	Yes - GN was a classmate of the participants (Methods: Interview Format)
7	Participant's knowledge of the interviewer	What did participants know about the researcher?	Participants knew GN was a fellow medical student researching POCUS education (Methods: Recruitment)
8	Interviewer characteristics	What characteristics were reported about the interviewer?	Peer status, insider familiarity with POCUS curriculum, potential for shared assumptions acknowledged (Methods: Interview Format)
Domain 2: Study Design			
Theoretical Framework			
9	Methodological orientation	What methodological orientation underpinned the study?	Constructivist; inductive thematic analysis (Methods: Study Design)
Participant Selection			
10	Sampling	How were participants selected?	Convenience sampling via email from a pool of 140 final-year students (Methods: Recruitment)
11	Method of approach	How were participants approached?	Email invitation with one reminder (Methods: Recruitment)
12	Sample size	How many participants were in the study?	13 (Methods: Recruitment)
13	Non-participation	How many people refused or dropped out? Reasons?	127 did not respond; reasons unknown. 9.3% response rate acknowledged. Self-selection bias discussed (Methods: Recruitment; Limitations)
Setting			
14	Setting of data collection	Where was the data collected?	Telephone interviews (Methods: Interview Format)
15	Presence of non-participants	Was anyone else present besides participants and researchers?	No (Methods: Interview Format)
16	Description of sample	What are the important characteristics of the sample?	Final-year MD students at Emory: 5 male, 8 female; ages 25–36; applying to 6 specialties (Results: Participant Demographics)
Data Collection			
17	Interview guide	Were questions, prompts, and guides provided by authors?	Yes - Table 3 (Methods: Interview Format)
18	Repeat interviews	Were repeat interviews carried out?	No (Methods: Interview Format)
19	Audio/visual recording	Did the research use audio or visual recording?	Audio recording via Voice Memo (Methods: Data Collection)
20	Field notes	Were field notes made during and/or after the interview?	Not systematically collected (Methods: Data Collection)
21	Duration	What was the duration of the interviews?	10-30 minutes (Methods: Interview Format)
22	Data saturation	Was data saturation discussed?	Yes - informally observed, no new themes in final interviews; formal threshold not used (Methods: Data Collection)
23	Transcripts returned	Were transcripts returned to participants?	No - member checking not performed (Methods: Data Collection; Limitations)
Domain 3: Analysis and Findings			
Data Analysis			
24	Number of data coders	How many data coders coded the data?	2 (GN and PN) with senior review by MH (Methods: Data Collection)
25	Description of coding tree	Did the authors describe the coding tree?	Three rounds of coding; broad categories → subcategories → refined schema (Methods: Data Collection)
26	Derivation of themes	Were themes identified in advance or derived from data?	Derived from data - inductive approach (Methods: Study Design & Data Collection)
27	Software	What software, if applicable, was used to manage data?	Microsoft Excel (Methods: Data Collection)
28	Participant checking	Did participants provide feedback on findings?	No - member checking not performed (Methods: Data Collection; Limitations)
Reporting			
29	Quotations presented	Were participant quotations presented? Was each quotation identified?	Yes, quotations are presented throughout the Results. Attributed broadly to the sample rather than the individual participant codes (Results)
30	Data and findings consistent	Was there consistency between the data presented and the findings?	Yes - themes and interpretations presented in the Discussion are grounded in and consistent with the participant quotations in Results. (Table 2)
31	Clarity of major themes	Were major themes clearly presented in findings?	Yes - 3 themes with subthemes presented; summary in Table 2 (Results)
32	Clarity of minor themes	Is there a description of diverse cases or minor themes?	Yes - dissenting views on timing presented; barrier of specialty relevance noted (Results: Theme 3)

Appendix 2 

**Table 4:** Semi-Structured Interview Guide Domain-organized interview questions were used to explore final-year medical students’ POCUS education experiences, attitudes, and instructional preferences. POCUS: point-of-care ultrasound.

Domain	Interview Questions
Demographics	1. What is your age? 2. What is your gender? 3. What specialty are you applying to?
Level of Exposure	1. What ultrasound exposure/training did you have before medical school? 2. What ultrasound exposure/training did you have during preclinicals? 3. What ultrasound exposure/training did you have during clinical rotations?
General Attitude	1. How important do you think it is to learn ultrasound knowledge/skills during medical school? 2. How comfortable are you with interpreting ultrasound images? 3. How comfortable are you with performing an ultrasound exam?
Method of Instruction	1. How have you been taught ultrasound theory/skills during medical school? 2. What were the most effective methods of learning ultrasound? 3. How would you have preferred to learn ultrasound?
Timing	1. When do you think ultrasound should be taught during the medical school curriculum?
Content	1. What ultrasound knowledge would have been useful to have learned during medical school? 2. What ultrasound skills would have been useful to have learned during medical school?
